# AMPK: a balancer of the renin–angiotensin system

**DOI:** 10.1042/BSR20181994

**Published:** 2019-09-03

**Authors:** Jia Liu, Xuan Li, Qingguo Lu, Di Ren, Xiaodong Sun, Thomas Rousselle, Ji Li, Jiyan Leng

**Affiliations:** 1 Department of Geriatrics, The First Hospital of Jilin University, Changchun 130021, China; 2 Department of Surgery, University of South Florida, Tampa, FL 33612, U.S.A.; 3 Mississippi Center for Heart Research, Department of Physiology and Biophysics, University of Mississippi Medical Center, Jackson, MS 39216, U.S.A.

**Keywords:** ACE, AMPK, Ang II, RAS

## Abstract

The renin–angiotensin system (RAS) is undisputedly well-studied as one of the oldest and most critical regulators for arterial blood pressure, fluid volume, as well as renal function. In recent studies, RAS has also been implicated in the development of obesity, diabetes, hyperlipidemia, and other diseases, and also involved in the regulation of several signaling pathways such as proliferation, apoptosis and autophagy, and insulin resistance. AMP-activated protein kinase (AMPK), an essential cellular energy sensor, has also been discovered to be involved in these diseases and cellular pathways. This would imply a connection between the RAS and AMPK. Therefore, this review serves to draw attention to the cross-talk between RAS and AMPK, then summering the most recent literature which highlights AMPK as a point of balance between physiological and pathological functions of the RAS.

## Introduction

The renin–angiotensin system (RAS) is one of the most prominent endocrine (tissue-to-tissue), paracrine (cell-to-cell), and intracrine (intracellular/nuclear) vasoactive systems [1]. Initially considered a regulator of the vascular system and an active proponent toward the development of hypertension, the RAS has developed extensively in recent years through its links to obesity, diabetes, and hyperlipidemia [2–6]. Parallel to the development of RAS in the literature, AMP-activated protein kinase (AMPK)—a stress-inducible energy sensor–has been highlighted as a coordinator of metabolism and has been intimately connected to all aspects of cellular function that are involved in RAS-related disease [7–10]. The blockade treatment of the classical RAS pathway has gained widespread recognition [6,11,12], with its proponents endorsing the suppression of the RAS to up-regulate the activity of AMPK. However, opponents of this theory imply that some AMPK activators are effective for RAS-related diseases and can affect the expression of RAS-related components. In this review, the cross-talk between RAS and AMPK will be discussed in terms of both physiological function and implications of dysfunction between these systems which leads to disease progression. With special consideration given toward the cardiovascular system, possible mechanisms will be discussed as well as the significance of these discoveries for future animal experiments and clinical research.

## A brief overview of RAS

In the year 1898, Tigerstedt and Bergman discovered renin to be the rate-limiting enzyme and the first recognized component of RAS [1]. At first, the RAS was thought to be a sequence of many enzymatic steps, eventually producing a single bioactive metabolite, Angiotensin II (AngII) [2,13]. However, in recent years, the identification of novel enzymes and receptors has magnified the classical view of the RAS [14,15]. Identifying the RAS as a system of two counter arms may be a milestone in the history of RAS research [15]. The two counter arms include the classical RAS path (Ang-converting enzyme (ACE)/AngII/Ang type 1 receptor (AT1R)) and the protective arm (ACE2/Ang 1-7/Mas receptor (MasR)) [14].

**Figure 1:**
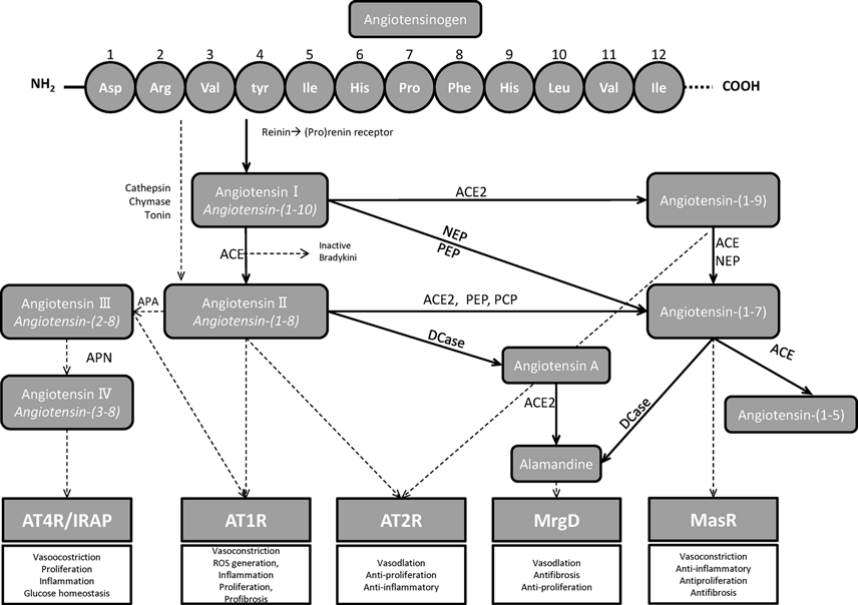
The overview of RAS cascade Abbreviations: AP, aminopeptidase; APA, aminopeptidase A; APN, aminopeptidase N; DCase, decarboxylase; PCP, prolyl carboxy endopeptidase; NEP, neutral endopeptidase; PEP, prolyl endopeptidase; MrgD, Mas-related G-protein-coupled receptor D.

The two arms, all start from the same point, angiotensinogen (Agt). The primary source of plasma Agt is in the liver. The first 12 amino acids -Asp-Arg-Val-Tyr-Ile-His-Pro-Phe-His-Leu-Val-Ile- are considered to be the most necessary for its biological activity. Prorenin and renin circulating in the blood are mainly produced in the juxtaglomerular epithelial (JGE) cells [16]. Renin is a highly specific enzyme; it acts on renin substrate- (Agt) and catalyzes the first step in the cascade of the RAS compounds. Working on Agt, renin cleaves the decapeptide angiotensin I (Ang I) to AngII by angiotensin-converting enzyme 1 (ACE, ACE 1). AngII is the core active peptide of the classical arm. Other enzymes can also participate in the production of AngII, such as cathepsin, chymase, and tonin [17]. Furthermore, ACE can inactivate bradykinin. Bradykinin is a potent vasodilator peptide, and it also presents beneficial effects to insulin-dependent glucose transport activity [18] (Figure 1).

AngII exerts physiological effects by combining with angiotensin II type 1 (AT1R) and type 2 (AT2R) receptors. AngII combines with AT1R to achieve most of its physiological effects [19,20]. AT1R is mainly located in kidney, liver, lungs, and vascular smooth muscle cells. On binding to AngII, also mean to activate the classical arm (ACE/AngII/AT1R), AT1R stimulates vasoconstriction, sodium retention, sympathetic nervous activation, and reactive oxygen species (ROS) generation, coupled with deleterious effects, including endothelial dysfunction and induction of inflammatory, thrombotic, proliferative, and fibrotic processes [14,19,21]. Furthermore, researchers discovered that AngII regulates the homologous by binding to AT1R and this effect is related to cell types [22–24]. AT2R is mainly expressed in fetal tissues while in the neonatal period its expression declines, and it expresses mostly in the kidney, heart, brain, and blood vessels [25]. The gene for AT2R is located on the X-chromosome [25], which has a low homology of amino acid sequence (∼ 34%) compared with AT1R [24]. In contrast with AT1R, AT2R appears to counteract AT1R in most cases, exerting tissue-protective effects which include vasodilation, natriuretic, anti-inflammatory, and anti-proliferative effects (Figure 1).

In the RAS protect arm, by activating ACE2, AngII is degraded to Ang(1-7), and this is the main way to generate Ang(1-7). Ang(1-7) can also be directly degraded from Ang I by other enzymes [26]. Alternatively, Ang I can be hydrolyzed to Ang1-9 by ACE2 and then Ang1-9 is cleaved to Ang(1-7) by activating ACE [15]. Through binding to MasR, Ang(1-7) triggers anti-inflammatory, anti-fibrotic, and anti-proliferative actions, in one world, opposing the actions accused by AngII [27,28]. Furthermore, newer pieces of evidence showed that Ang1-9 has beneficial effects specially in preventing/ameliorating cardiovascular [29–31]. The beneficial effects are not only through Ang(1-7) pathway, but also directly mediated via the AT2R, highlighting that Ang1-9, along with Ang(1-7), makes up part of the counter-regulatory arm of the RAS [32–34]. At the same time, more studies about RAS have discovered new peptides and receptors, but their biological effects have not been fully explored [1,16]. Several new pathways of the RAS were discovered. For instance, the AngII/ aminopeptidase A (APA)/AngIII/AT2/NO/cGMP [35], the AngIII/ aminopeptidase N (APN)/AngIV/IRAP/AT4 receptor [35], and the AngII/ACE2/Ang(1-7)/Almamndine/Mas-related G-protein-coupled receptor D (MrgD) receptor (Figure 1) [36,37]. At the same time, local RAS components also caught the attention of the researchers. The local RAS have been discovered in almost every tissue, for example, heart, kidney, blood vessels, and so on [17], and also played an important physiological role.

## A brief review of AMPK

AMPK is a serine/threonine protein kinase, which is regarded as an energy change sensor in the cell and modulator of metabolic responses to maintain energy homeostasis [38,39]. It has been studied for nearly 50 years since its first discovery [7,14,40–42]. The structure of AMPK includes a catalytic α subunit and regulatory β and γ subunits. Seven distinct genes code (α1/α2; β1/β2; γ1/γ2/γ3) for the various isoforms of AMPK [40,43–45]. The catalytic domain of α subunit promotes the translocation of a phosphate group from adenosine triphosphate (ATP) to the Ser/Thr sites. The N-terminus of α subunit is active only after phosphorylation in the activation loop at Thr^172^, which is now widely used as a biomarker for AMPK activation [9,43]. The β subunit provides a structural bridge between α and γ subunits by a carbohydrate-binding module (CBM) connected to the glycogen [9]. The γ subunit is allosteric activator; its regulatory function is acted through CBS sites 1 and 3, which can bind to AMP, ADP, or ATP. Through AMP binding to site 1, the enzyme is activated. At site 3, binding of either AMP or ADP will suppress the ability of phosphatases to remove the phosphate from Thr^172^ and inhibit the enzyme [9,43].

The phosphorylation of AMPK is largely dependent on the AMP and ATP ratio [43,46–49]. After AMP binding to the AMPK γ-subunit, the allosteric structure will change. Because of this change, the AMPK activity will be elevated two- to five-folds [49]. AMPK is also significantly regulated by upstream kinases. The AMPK activity will be elevated to hundreds of folds by phosphorylating the Thr^172^ [49,50].

Up to now, three kinases and three phosphatases have been discovered as upstream AMPK-activating kinases. The three kinases contain Liver kinase B1 (LKB1), Ca^2+^-/calmodulin-dependent protein kinase β (CaMKKβ), TGFβ-activated kinase 1 (TAK1). The three phosphatases include protein phosphatase 2A (PP2A), protein phosphatase 2C (PP2C), and Mg^2+^-/Mn^2+^-dependent protein phosphatase 1E (PPM1E) [48,51]. AMPK is also activated in several physiological and pathological conditions, such as hypoxia, caloric restriction (CR), and physiological exercise. Furthermore, the intracellular calcium, oxidant signaling, and extracellular signalings like hormones and cytokines also can modulate the AMPK activity [52]. AMPK is also the target of many pharmacological activators [53,54]. Several factors can inhibit the activity of AMPK; it includes high glucose and glycogen, lipid overload, amino acids [47], and pharmacological AMPK inhibitor [47,55]. AMPK is widely located in various cells and organs [44,45]. In many pathological conditions, the body adapts to these challenges by activating AMPK, which modulates numerous downstream targets [47]. The concept that the activation of AMPK is beneficial to various diseases has been widely recognized [56–58]. It has been proved that activating AMPK can improve cardiometabolic disease [7], protect from myocardial ischemia [7], inhibit cardiac hypertrophy and cardiomyopathy [8,59–61], protect heart function and delay heart failure [7,62–65], and antiarrhythmia [10]. Additionally, the decreased activity of AMPK has also been shown to participate in hypertension [48,66], lipid metabolism, inflammation [58], obesity, insulin resistance, type 2 diabetes [67], renal pathophysiology, aging [68], and tumor [68,69]. At the same time, complex cross-talk between AMPK and other cell cytokines and signaling pathways, for example, sirtuins, the insulin/IGF1, Ras-Raf-MEK-ERK pathways have attracted interests [70]. Although some experiments have investigated the possible links between RAS and AMPK, the clear mechanism is still controversial.

## The cross-talk between RAS and AMPK

**Figure 2:**
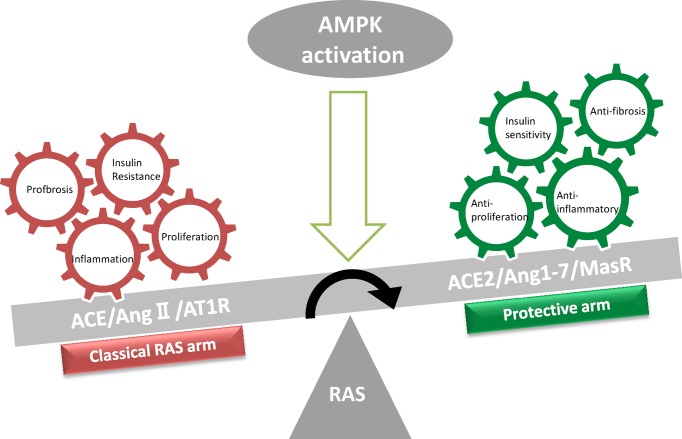
Schematic representation of RAS modulation following AMPK activation When the RAS is activated, the balance between the classical RAS arm (ACE/AngII/AT1R, red frame) and the protective arm (ACE2/Ang 1-7/MasR, green frame) is broken, thus the pathological changes of the body are induced and concentrated. At this situation, when the AMPK activator is given, the expression of ACE and AT1R are suppressed, while the expression of ACE2 and MasR are increased. At the same time, the up-regulated ACE2 increases the metabolism of AngII to Angl-7, thus activation of AMPK inhibits the classical RAS pathway, and elevation of the RAS protection arm, which keeps the RAS balance.

Because RAS is a community of many factors, each component is interconnected and mutually constrained. The current research is mainly about the classic arm and the protective arm we mentioned earlier. In this part, we mainly focus on the relation between the factors involved in these two arms and AMPK (summarized in Table 1). Finally, the two arms as one system and their correlation with AMPK will be discussed (Figure 2).

**Table 1:** The possible relationship between RAS components and AMPK

Components of RAS	Tissue or cell type	Relation to AMPK		Related studies
ACE	Heart	No direct relation was shown		[77–79]
	Vascular system endothelial cells	AMPKα2 suppresses endothelial ACE expression via the phosphorylation of p53 and up-regulation of miR-143/145		[75]
	Monocytes	ACE expression was reduced in spleen derived-monocytes from AMPKα1(−/−) mice versus their wild-type littermates		[74]
	White adipose tissue	AMPK may decrease ACE expression		[76]
AngII	Heart	Rat neonatal cardiomyocytes	AngII down-regulates AMPK (α-Thr^172^) may be by phosphorylating α-Ser^485/491^ or inhibiting LKB1	[78,84–86]
		Neonatal rat ventricular myocytes (NRVMs)	Activation of AMPK can ameliorate pathological damage induced by AngII	[103,108]
		H9C2 cells neonatal rat cardiomyocyte (NRCMs)	No direct relation was shown	[88,89]
		HL-1 atrial myocytes	Ang ІІ decreased oxygen consumption rate, which resulted in ROS generation, AngII-induced intracellular calcium production. The generated ROS and calcium stimulated AMPK phosphorylation. Inhibiting AMPK blocked AngII-mediated JNK and TGF-β signaling pathways	[90]
AngII	Vascular system	VSMCs	AngII-induced AMPK activation and that AMPK works as an inhibitor of the AngII proliferative pathway	[91]
		VSMCs	AngII led to minor activation of AMPK at a low concentration (0.1–1 μM), whereas AngII suppressed AMPKα activity at a high concentration (5 μM) AngII could elevate LKB1 expression in VSMCs, while the activity of LKB1 was not elevated with the expression of LKB1, even declined slightly	[92–94]
		VSMC human umbilical vein endothelial cells (HUVECs)	Activation of AMPK can ameliorate pathological damage induced by AngII	[104–107,109]
AngII	Skeletal muscle	AngII inhibits AMPK Thr^172^ and AngII might up-regulate the AMPK resistance		[97,109]
	Kidney	AngII inhibits AMPK by binding to AT1 p-AMPK increase after ARB application		[100–102,105]
AT1R	Heart	AT1 is negatively related to AMPK, ARB could phosphorylate AMPK		[112,113,117]
	Skeletal muscle	ARB can improve AMPK resistance and activate AMPK		[97–99]
AT2R	White adipocytes	AT2R may activate AMPK independent of AngII		[119]
	Pulmonary artery endothelial cells (PAECs)	By binding to AT2R, AngII induces apoptosis by phosphorylating AMPK β		[120,121]
	Chinese hamster ovary (CHO)-K1 cells	Both AT1R and AT2R overexpression activated AMPK		[120]
ACE2	Heart	ACE2 could activate AMPK		[85,123,124]
	Adipose tissue	Phosphorylation of AMPK is reduced when ACE2 is knocked out		[124]
	Huh7 (hepatocellular carcinoma-derived) cells	AMPK activation can increase ACE2 expression, but this effect requires sirtuin 1 (SIRT1) to participate		[76,125]
	HUVECs and human embryonic kidney 293 (HEK293T) cells	Phosphorylated ACE2 Ser^680^ by AMPK could enhance the ACE2 stability		[126]
Ang(1-7)	Vascular systemaortas versus aortic tissues	Ang(1-7) could activate AMPK		[124,131]
	White adipose tissue	Ang(1-7) could activate AMPK		[76]
MasR	Adipose tissue	MasR may be positively related to AMPK		[76,124]
MrgD	Ventricular cardiomyocytes	Almandine could activate AMPK by activating LKB1		[89]

### ACE and AMPK

The ACE is critical in the generation of angiotensin II (AngII) [71], it plays the main role in the classic RAS pathway [72]. AMPK has been proved to associate with many kinds of diseases, for instance cardiovascular disease [9,63], metabolic syndrome [56,67], renal pathophysiology [73], and aging [44]. So there should be some association between ACE and AMPK.

Based on recent studies, it seems that AMPK could regulate the expression of ACE. Increased phosphorylation of AMPK could suppress the expression of ACE. However, this effect may be tissue or cell type dependent. In 2011, Kohlstedt et al. [74] discovered that the mature adipocytes modulated the expression profile of macrophages and the process involved increased ACE expression via AMPKα. In their further study (2013), they confirmed the AMPK-p53–miR-143/145-ACE pathway both *in vivo* and *in vitro* [75]. In another study, the researchers observed the increased activity of AMPK and decreased expression of ACE in white adipose tissue by oral administration of Ang(1-7) and resveratrol to high-fat diet (HFD) mice [76].

While up to now, the studies in heart present little relation between ACE and AMPK. In an isolated heart model study, captopril (an ACEI) did not show the ability that it can phosphorylate AMPK [77]. In another study, in the heart tissue of SHRs, the results showed that activation of AMPK might be unrelated to ACE [78]. Although based on the research of Suarez-Martinez [79], by scoring the staining intensity of AMPK in the heart, AMPK was more abundant in enalapril (an ACEI) treated group while less in the ApoE-deficient control group.

### AngII and AMPK

AngII is considered to be the finical production of the classic RAS [2,13]. It has a direct role in the cardiovascular, damage of the glucose transport system [3], and insulin resistance [80]. It also participates in proliferation, hypertrophy, autophagy, and apoptosis [81–83]. Meanwhile, AngII leads to severe impaired cell signaling, which is also affected by AMPK [10]. Different functions were shown by AngII in different types of cells [22]. There may be some differences among different cells about the relationship between AngII and AMPK.

#### AngII and AMPK in heart

AngII may suppress the activation of AMPK in cardiomyocytes, which is related to influencing the downstream cell pathway of AMPK, phosphorylating Ser^485^ (α1)/Ser^491^ (α2) site of AMPK and down-regulating expression of LKB1 (an upstream kinase of AMPK). In 2008, Stuck et al. [84] learned that by inhibiting AMPK, AngII induced hypertrophy of cardiomyocytes. This process involved up-regulating glucose uptake and down-regulating mTOR. Furthermore, they discovered that the down-regulation of AMPK by AngII had a time- and concentration-dependent manner. But they did not explain how AngII suppresses the activity of AMPK [84]. Another study confirmed this effect [85]. In 2010, Jiang et al. [86] discovered that the α-Thr^172^ phosphorylation site of AMPK is inhibited following increased α-Ser^485/491^ phosphorylation site of AMPK in the setting of AngII-induced cardiac hypertrophy [86]. There is a counter cross‐talk between AMPKα-Ser^485/491^ and AMPKα-Thr^172^ [87]. In a further study, Zhu et al. (2014) [78] found that ventricular hypertrophy induced by AngII in SHRs might be related to inhibiting LKB1/AMPK activity. But not all the experiments supported this finding. Chen et al. [88] reported that when H9C2 cells were co-cultured with 5 μM of AngII for 12, 24, or 48 h, autophagy was enhanced, while AMPKα/mTOR signaling or cellular ADP/ATP ratio was not impacted. In another study, neonatal rat cardiomyocytes (NRCMs) were treated with AngII (100 nmol/l) for 36 h. The results showed that AngII did not affect the phosphorylation of AMPK [89]. Kim et al. [90] claimed that by activating the AT1R-calcium-AMPK-JNK axis pathway, AngII induces inflammation in atrial cells. But in their experiment, they only studied the AMPK inhibitor. While they did not give AMPK activator such as AICAR to confirm whether extra active AMPK will deteriorate the inflammation caused by AngII in atrial cells [90]. Further studies are needed.

#### AngII and AMPK in the vascular system

AngII plays a crucial role in the vascular system. AMPK is also proved to participate in the regulation of the vasculature. Based on current experimental data, AngII stimulated proliferation, migration, inducing ER oxidative and stress in vascular smooth muscle cells may partly depend on inhibiting expression of p-AMPK. AngII may also suppress the activity of LKB1. But this effect seems to have a time- and dose-dependent manner. In 2004, Nagata et al. [91] first reported that AngII (10^−7^ mol/l AngII for 30 min) activated AMPK in rat VSMCs via the AT1R-NADPH oxidase-O_2_-H_2_O_2_ axis. And AMPK, which is activated by AICAR, could inhibit AngII-induced MEK/ERK activation. While they did not detect the RAS components in this experiment. Later studies showed that when the treating time and concentration are sufficient, AngII tended to suppress the activity of AMPK [92,93]. There was another study, which declaimed AngII could elevate LKB1 expression in VSMCs and neointima. While the activity of LKB1 was not elevated with the expression of LKB1, it even declined slightly [94].

#### AngII and AMPK in skeletal muscle

The overactivation of RAS played a significant role in the process of insulin resistance in skeletal muscle [95,96]. Several studies have supported that AngII could induce insulin resistance in skeletal muscle. Akt signaling and insulin-mediated glucose transport were injured by AngII treatment in rat skeletal muscle and these signalings were also affected by AMPK [4]. The recent studies show that AngII suppresses the activity of AMPK not only by down-regulating the phosphorylation of AMPK [97,98] but also by up-regulating the AMPK resistance in skeletal muscle [99].

#### AngII and AMPK in kidney

Current research supports that AngII inhibits AMPK activity through AT1R in the kidney. Deji et al. (2012) [100] claimed that Ang-II down-regulated AMPKα phosphorylation in the kidney resulting in altered sodium retention and enhanced salt-sensitivity. The expression of phosphorylated AMPKα is improved when losartan is administered [100]. In another study, researchers claimed that AngII led to the relocation and down-regulation of podocyte AMPKα via AT1R and MAPK signaling pathway [101]. In further studies, Ha et al. [102] found that AngII could influence the distributional and quantitative effect of podocyte p130Cas and this effect was mainly dependent on the inhibition of AMPK.

In addition to direct observation of AMPK changes after treatment with AngII, there are many experimental studies demonstrating that activation of AMPK can ameliorate pathological damage induced by AngII. One study found that globular adiponectin (gAd) inhibits AngII‐induced NF-κB activation in hypertrophic neonatal rat ventricular myocytes (NRVMs) via AMPK activation [103]. Another study reported that losartan activated AMPK activation, inhibited the AngII-induced VSMC proliferation by cell cycle arrest [104]. Another research claimed that adiponectin weakens AngII-induced oxidative stress through cAMP-Epac and AMPK signal pathways in renal tubular cells [105]. Cao et al. [106] studied cultured human vascular smooth muscle cells (hVSMCs), and discovered that the activation of AMPK by resveratrol inhibited AngII-induced phosphorylation of myosin phosphatase-targeting subunit 1 (MYPT1) and myosin light chain (MLC); Liraglutide, a GLP-1 receptor agonist, may inhibit VSMC proliferation which is induced by AngII. This is carried out by activating AMPK signaling and causing cell cycle arrest [107]; Baicalin prevented the development of cardiac fibrosis, mediated by AngII, by activating AMPK/TGF-β/Smads signaling pathway [108]; restoration of AMPK activity during AngII-induced hypertension ameliorates vascular function which was related to inhibition of NADPH-oxidase and xanthine oxidase activity. This result suggested AMPK as a possible pharmacological target in vascular disease [105]; AngII-induced human umbilical vein endothelial cells (HUVECs) senescence was improved by the apelin/APJ (one G protein-coupled receptor with seven transmembrane domains) axis via the AMPK- sirtuin 1 (SIRT1) signaling pathway, and the mechanisms might be associated with the suppressed production of ROS and the enhanced activity of telomerase [109].

Altogether, most studies *in vivo* supported that AngII suppresses the activity of AMPK via AT1R, this is also confirmed *in vitro*. In Nagata et al.’s [91] research, they found that AngII could activate AMPK, but the test was performed within 1 h, and if they extend the duration of action, will the results change? In another study, low concentrations (0.1–1 μM) of AngII led to minor activation of AMPK, whereas high concentrations (5 μM) of AngII decreased AMPKα phosphorylation in human VSMCs [93]. We hypothesize that AngII activates AMPK at a small initial dose and protects it. When the AngII dose or treatment time continues to increase, it may inhibit AMPK activity and cause cell damage. According to Kim et al.’s [90] study, AngII induces inflammation through activating the AT1R-calcium-AMPK-JNK axis pathway in atrial cells. Active AMPK may be harmful. It may suggest that the association between AngII and AMPK also has tissue and cell dependence. There are not many studies that show how the AngII level changes during activation of AMPK, while several studies show the changes of AT1R or AT2R after administration of AMPK activator. We will review it in the next section.

### AT1R, AT2R, and AMPK

In the previous section, we discussed the relationship between AngII and AMPK. At the same time, as receptors for AngII, AT1R, and AT2R’s relationship with AMPK is also concerned. Many ARBs (AT1R suppressor) have shown the function of activating AMPK [110,111]. This proves that AT1R has some connection with AMPK. As the researchers deepened their understanding of AT2R, they also attracted scholars’ interest in research on AT2R. In this part, we will discuss the cross-talk among AT1R, AT2R, and AMPK.

#### AT1R and AMPK

##### AT1R and AMPK in heart

Based on several studies, AT1R and AMPK may have a negative reciprocal relationship in the heart. Up-regulation of AT1R may suppress the activity of AMPK. But the exact mechanism is unknown. According to Hernández et al.’s [112] study, a negative reciprocal connection between AMPK activation and AT1R levels in AngII-induced cardiomyocyte hypertrophy was reported. By using a transgenic rat model, the researcher announced that the activated RAS might inhibit AMPK by up-regulating AT1R [113]. In Vázquez-Medina et al.’s study, they found that ARB treatment could improve damaged insulin signaling and maintain cellular energy balance during metabolic syndrome by increasing AMPK activity in the heart [114–116]. In another research, the phosphorylation of AMPK showed no differences between Long Evans Tokushima Otsuka (LETO) group and Otsuka Long Evans Tokushima Fatty (OLETF) group. But phosphorylation of AMPK raised 60% after ARB administration in OLETF versus control group [117].

##### AT1R and AMPK in skeletal muscle

There is no direct relation between AT1R and AMPK according to recent experiments, but three experiments studied with ARB (AT1R blocker) were performed. Shinshi et al.’s [99] study found that acute inhibition of AngII receptors does not improve insulin resistance, but rather improves AICAR resistance [118]. In the further study, Yoshida et al. [97] found that AICAR resistance was improved by an ARB chronic treatment, this process was related to GLUT-4. Based on the study of Lastra et al. [98], insulin signaling in the red gastrocnemius muscle of AngII treated rats can be improved by long-term (8 weeks) administration of AZIL-M (ARB). This effect may be related to increased phosphorylation of AMPK and decreased phosphorylation of the signaling pathway of p70S6K1 [98].

#### AT2R and AMPK

Studies on the relationship between AT2R and AMPK extended mainly in adipose tissue and lungs. One investigation revealed that in white adipocytes, AT2R may activate AMPK independent of AngII [119]. AT2R induced white adipocyte browning by increasing peroxisome proliferator-activated receptor (PPARγ) expression in primary cultures of mouse white adipocytes, which at least partially passes through AMPK, PI3k/Akt and ERK1/2 signaling pathways [119]. Another research demonstrated that AngII adjusts the relationship of AMPKβ1/2 between Cdk4, leading to the hyperphosphorylation of retinoblastoma protein (Rb) and induction of E2F1(E2F is a group of genes that encodes a family of transcription factors in higher eukaryotes, E2F1 is one activator of the family)-dependent Bim gene activation in pulmonary artery endothelial cells (PAECs) [120,121]. Day et al.’s [120] lab proposed that AngII-induced ATP production via activation of AT2-AMPK pathway, and is required for apoptosis in PAECs. In their further study, they concluded that AngII regulates the association of AMPKβ1/2 with Cdk4 via AT2R, leading to the hyperphosphorylation of Rb and induction of E2F1-dependent Bim gene activation [121].

AT1R and AT2R are both components of classic RAS [19,21]. There are three possibilities for why ARB, which is closely related to AT1R and AT2R, can activate AMPK. The first, it depends on the activation of this compound to effectively abolish the action of AngII. The second, ARB could block the AT1R. As a result, the AngII level is up-regulated, then the increased AngII may participate in the regulation of AMPK via AT2R. The last is that some ARBs may activate AMPK through another way independent of AT1R or AT2R. Combining with the previous chapter, we have reason to believe that the classical RAS pathway is related to AMPK, furthermore, they might be negatively related.

### ACE2 and AMPK

ACE2 is a zinc metalloproteinase consisting of 805 amino acids with significant homology to ACE1, but it is not inhibited by ACE1 inhibitors [26]. As a core enzyme in the metabolism of AngII, ACE2 directly regulates the levels of AngII and Ang(1-7). ACE2 is accepted as an Ang(1-7) marker and considered to be a feasible therapeutic target of the RAS [26,122]. Based on current researches, ACE2 and AMPK have closed relations.

#### ACE2 increases the activity of AMPK

According to Murça et al.’s [123] study, ACE2 regulates AMPK activity to improve cardiac metabolic imbalance and ameliorates the outcomes in diabetes-induced cardiac dysfunction. It involves the regulation of AMPK-α and β1 and inhibition of ERK. Diabetic rats were orally administered with ACE2 activator or saline (control) for 30 days. The diabetic animals treated with saline showed a decreased Mas/AT1R receptor protein expression ratio, a down-regulated AMPK-α activation along with an up-regulated AMPK-β1 activation and augmentation in the AT2R protein expression. In addition, there was no interference with AT2R expression, but treatment with the ACE2 activator 1-[[2-(dimethylamino ethyl] amino-4-(hydroxymethyl)-7-[[4 methylphenyl)sulfonyl]oxy]-9hxanthen-9-one(XNT) prevented these changes [123].

#### ACE2 knockout reduces the activation of AMPK

In another study, ACE2 null (ACE2KO) and wild-type (WT) mice were treated with a HFD or a control diet and studied at 6 months of age. Researchers found that a shortage of ACE2 aggravates epicardial adipose tissue inflammation and cardiac dysfunction in the diet-induced obesity model. Decreased AMPK activation may have contributed to this effect. Activation of AMPK was up-regulated in WT-HFD hearts, whereas down-regulated in ACE2KO-HFD hearts. There was no significant difference in the protein levels of MasR, AT1R, and ACE in WT and ACE2KO hearts [124]. In Zhang et al.’s [85] study, the reduced levels of SIRT6, p-AMPKα, the increased expression of FKN and plasma AngII level was observed in ACE2KO mouse hearts. After administration of sirtuin6, AMPK phosphorylation and ACE2 expression were both elevated [85].

#### AMPK may increase the expression of ACE2

In Andrade et al.’s [76] study, they found that Ang(1-7) and resveratrol can improve glucose metabolism through AMPK/FOXO1/PPAR-γ pathway, while, both drugs could increase the expression of ACE2, SIRT1 and reduce the expression of ACE in adipose tissue. When Mas antagonist A779 and Sirtinol antagonists were given to each group, the effects were blocked. This indicated that the blockade or activation of sirtuins (Sirtinol/resveratrol) regulates the expression of RAS components and the blockade or activation of RAS/Ang(1-7) axis [A779/Ang (1-7)] regulated the expression of sirtuins. It is interesting that at this progression, both Ang(1-7) and resveratrol could active AMPK [76].

##### AMPK regulates the expression of ACE2

One study found that activation of AMPK induces an increase in ACE2 expression, and SIRT1 was involved in this process [125]. Both AICAR and metformin could activate AMPK, but only AICAR could up-regulate ACE2. It might be due to AICAR that can activate SIRT1, while metformin does not have this effect. SIRT1, in the presence of a possible unknown cofactor, binds to the promoter region of ACE2 and this binding is promoted by AICAR. Interestingly, an inhibitor of SIRT1 also could inhibit the ACE2 expression caused by AICAR. This strongly supports that the SIRT1 may be related to the regulation of ACE2 under conditions of energy stress [125].

##### AMPK increases the stability of ACE2

In a recent study, researchers found that AMPK could phosphorylate ACE2 Ser^680^ in HUVECs and human embryonic kidney 293 (HEK293T) cells [126]. Phosphorylated ACE2 Ser^680^ by AMPK could enhance ACE2 stability and the bioavailability of NO [126]. This process is a crucial mediator of PAEC function. The core mechanism is related to the cross-talk between phosphorylation and ubiquitination, which could enhance the stability of ACE2. Inhibition of this post-translational modification-dependent AMPK–p-ACE2 axis aggravates pulmonary hypertension (PH). This pathway might be a potential target for PH [126].

### Ang(1-7) and AMPK

Ang(1-7) is mainly produced from AngII by the enzyme ACE2. It is expressed both in circulation and local tissues [127,128]. By binding to the receptor Mas, Ang(1-7) acts as a counter-regulator to the actions of AngII [127,128]. Ang(1-7) could improve diastolic dysfunction in the db/db type 2 diabetic murine model. The mechanism involves suppressing cardiac hypertrophy, lipotoxicity, and adipose inflammation, as well as elevating the adipose triglyceride lipase [128–130]. The activity of AMPK also was involved in these pathological processes.

Ang(1-7) seems to have the ability to increase the activation of AMPK. Andrade et al. [76] found that Ang(1-7) could improve the AMPK/FOXO1/PPAR-γ pathway in white adipose tissue. Another study founded that Ang(1-7) acute stimulation activates AMPK in aortas versus the control group in both basal and pathological conditions. And the administration of Ang (1-7) to ACE2KO-HFD (ACE2 null mice were fed an HFD) mice led to decreased EAT inflammation as well as reduced cardiac lipotoxicity and steatosis. This may partly be attributed to the up-regulation of p-AMPK by Ang(1-7) [124]. According to research by Karpe and Tikoo [131], insulin-resistant rats fed HFD showed attenuation of Ang(1-7). The corresponding effects induced by Ang(1-7), such as vasodilation, endothelial nitric oxide synthase (eNOS) phosphorylation, AMPK, SIRT1, ACE2, and MasR expression, were also attenuated. Interestingly, heat shock (HS) prevented this attenuation [131]. Based on the above experiment, we could at least infer that there seems to be a positive correlation between Ang(1-7) and AMPK.

### Mas or MrgD receptor and AMPK

MasR is the particular receptor for Ang(1-7) [1], also a G-protein-coupled receptor, playing an important role in mediating Ang(1-7) triggered anti-inflammatory, antifibrotic, and antiproliferative actions, opposing the effects triggered by AngII [27,105,132–135]. Current experiments show that the Ang(1-7) activates AMPK through binding to the MasR [76,124]. Whether the MasR can activate AMPK independent of Ang(1-7) has not been reported.

MrgD, another receptor of RAS, was reported to participate in and mediate alamandine through the activation of AMPK [1]. Alamandine, a new heptapeptide discovered by Lautner et al. [37], is a new component in the RAS. Alamandine acts on the MrgD and expresses similar effects to Ang (1-7) [128,136]. Recent research claimed that alamandine could activate AMPK in ventricular cardiomyocytes via MrgD [89]. Alamandine also induced a significant increase in LKB1 phosphorylation. This occurred dependent on MrgD receptor, not MasR [89].

### The balance of RAS and AMPK

In the above chapters, we have discussed the correlation between various organizational factors of RAS and AMPK (summarized in Table 1), but we know that RAS is not a system with simple combinations of multiple elements. On the contrary, RAS is an organic whole. The elements are closely related, mutually restrictive, maintaining dynamic balance. At the moment, it is clear that AngII acts on AT1R and AT2R, also possibly the Mas and MrgD receptors, to play very critical roles in maintaining homeostasis [1]. It is an extremely complicated process. The RAS classic arm (ACE1/AngII) and the RAS counter-regulatory arm (ACE2/Ang(1-7)) [14] seem to play important ‘YIN’ and ‘YANG’ counter-regulatory roles in maintaining healthy cardiovascular, blood pressure, insulin resistance, metabolism, and renal function, also in the development of cardiovascular, hypertensive, diabetes, obesity, and renal diseases [1,11,12,76,96,137–140]. AMPK, an energy sensor and the regulator is also involved in the regulation of the internal environment and the above diseases. Therefore, researchers have gradually noticed the contact between RAS balance and AMPK, not just the relationship between RAS single component and AMPK.

Several studies focus on the relation between RAS balance and AMPK. In one direct study, researchers discovered uninephrectomy induces chronic renal impairments accompanied by persistent RAS activation and AMPK inhibition. This effect could generally be improved by RAS blockade with ACEI or ARB [141]. In another study, it is reported that local RAS is altered in the aging aorta through the activation of the prorenin receptor (PRR)–ACE–AngII–AT1R axis and inhibition of the ACE2–MasR axis [142]. In further studies, the results showed that in the aorta, resveratrol inhibited the expression of PRR and ACE and increased the expression of ACE2, AT2R, and MasR [143]. Besides, resveratrol down-regulated the serum AngII level and up-regulated Ang(1-7) level. *In vitro*, the VSMC experiment further confirmed these findings [143].

Furthermore, some studies also provide indirect evidence that RAS balance is associated with AMPK. Most of these studies were in the cardiovascular system. According to a study by Lakshmanan et al. [113], increased expression of AT1R and decreased expression of MasR were observed in the transgenic (Spontaneous Diabetic Torii, SDT) rats. At the same time, the phosphorylation of AMPK was suppressed [113]. According to Murca et al.’s [123] study, oral administration of an ACE2 activator could elevate Mas/AT1 receptor protein expression ratio and increase in the AMPK-α phosphorylation along with a decreased AMPK-β1 phosphorylation in the STZ group compared with control. But the results showed no obvious differences in the expression of AT2R in each group [123]. In Na et al.’s [144] study, vinegar and acetic acid decreased serum renin and ACE activities, AngII and aldosterone concentration in SHRs, simultaneously, elevated AMP/ATP ratios and the phosphorylation of AMPK, PPARγ, co-activator-1α (PGC-1α) [144]. In 2017, one study demonstrated that ACE2 overexpression down-regulated the mortality of rats with doxorubicin-induced cardiomyopathy [122]. Its mechanism may involve activation of the AMPK and PI3K-AKT pathways, inhibition of ERK pathway and TGF-β1 expression [122]. At the same time, ACE2 overexpression can reduce ACE expression, myocardial AngII levels, and increase myocardial Ang (1-7) degrees.

Based on the above experimental studies, we can speculate that there should be a potential cross-talk between the cardiovascular system RAS and the AMPK pathway. Under pathological conditions, when RAS is activated, the classical arm (ACE1/AngII) predominates. At this time, the RAS protective arm (ACE2/Ang(1-7)) receives inhibition, and AMPK activation is inhibited. When the RAS guard arm (ACE2/Ang(1-7)) is boosted, AMPK is activated, and the RAS classical arm is inhibited. Similarly, when AMPK is activated, the RAS protection arm is enhanced, the RAS classical pathway is inhibited, as a result, the RAS balance is promoted (Figure 2).

### Potential links between AMPK and RAS

RAS and AMPK may interact with each other through direct interactions, and current research suggests that the related roles may also involve some necessary factors. So far, at least three factors have attracted the attention of researchers.

#### Sirtuins

Sirtuins are a family of histone deacetylases that contain seven enzymatic activities in mammals (SIRT1–SIRT7) [145]. It has been identified as having a key role in regulating mitochondrial function, cardiac energy metabolism, and other diseases [146–148]. It has been reported that several subtypes of sirtuins having the potential to regulate AngII actions, cardiac energy metabolism, or both [149,150]. Sirt 1, which is ubiquitously expressed and the most studied sirtuin, controls blood pressure through inhibition of AT1R [149] and eNOS-dependent pathways [151]. Changes in the RAS system also affect the expression of sirtuins. AngII down-regulated the expression of Sirt1 [152]. Oral administration of Ang-(1-7) and resveratrol improved metabolic profile through a cross-modulation between RAS and sirtuins [76]. The correlation between sirtuins and AMPK was also be reported in the experiments [153,154]. Many studies have shown the important role of sirtuins in the relationship between RAS and AMPK which we have already mentioned before [76,85,109,112]. In Clarke et al.’s [125] study, they found that both AICAR and metformin could activate AMPK, but only AICAR could up-regulate ACE2. It might due to AICAR can active SIRT1, while metformin does not have this effect. This strongly supports that the SIRT1 may be related to the regulation of ACE2 under conditions of energy stress [125]. The interaction mechanism of action among RAS, AMPK, and Sirt1 in different tissues, cells, and various pathological conditions still needs further study and confirmation.

#### Adiponectin

Adiponectin is mainly secreted by fat cells, and its concentration in the circulatory system is very high, accounting for approximately 0.01% (approximately 3–30 μg/ml) of total human serum protein, and approximately 0.05% of rodents [155–157]. A growing number of studies have reported that adiponectin exerts multiple biological effects in different tissues or organs by binding to their receptors [156]. Adiponectin is closely related to the cardiovascular circulatory system [158,159], which is regulated by RAS. Some researches showed that Ang(1-7) supplement significantly increases the levels of adiponectin [28,160], not only in circulation but also in the myocardial [161]. At the same time, more studies in recent years have reported that adiponectin may achieve its protective effect by activating AMPK [162–165]. Adiponectin or adiponectin receptors agonists activate several downstream signal transduction pathways mediated by AMPK and PPAR-α to improve fibrotic disorders [156]. Studies have shown that adiponectin may play an essential role in the relationship between RAS and AMPK. On the one hand, adiponectin improves the adverse effects caused by RAS activation by activating AMPK [103–105]. On the other hand, Ang(1-7) could activate the AMPK by both adiponectin-dependent and -independent pathways [166–169]. But more research is still needed.

#### PPARs

PPARs are the members of the nuclear hormone receptor family. They are ligand-activated transcription factors, which are responsible for regulating gene expression [170,171]. There are three isoforms: PPARα, PPARβ, and PPARγ [172]. PPARγ is the most extensively studied. Some ARBs can activate PPARs and have been widely recognized [173–177]. Current research shows PPARs and AMPK are also closely related. PPARs may activate AMPK [178–180]. One study claims that PPARγ agonists enhanced AMPK phosphorylation [179]. The other finding suggests that micheliolide ameliorates liver steatosis by upregulating PPAR-γ expression, thereby inhibiting NF-κB-mediated inflammation and activating AMPK/mTOR-dependent autophagy [180]. In Lu et al.’s [178] study, by using GW9662 (inhibitor of PPARγ) and compound C (inhibitor of AMPK), they suggested that AMPK might be a downstream effector of PPAR-γ. It seems that AMPK also affects the expression of PPARs. In one study, researchers found that hispidulin increased the expression and transcriptional activation activity of PPAR-γ mediated by AMPK and ERK, not JNK [181]. Therefore, PPAR may be an essential factor in RAS and AMPK, but some studies have confirmed that some ARBs may activate AMPK through the CaMKKβ pathway [182]. In summary, ARB activates AMPK, but the exact activation mechanism is still unknown. It may be performed in a dependent or non-dependent PPAR manner. In short, the role of PPAR in the relationship between AMPK and RAS needs further study.

## Conclusions and perspectives

Altogether, AMPK may be a regulator of RAS. AMPK may be a potential target for the regulation of RAS. Under physiological conditions, RAS keeps the balance, and plays a significant role in the maintenance of homeostasis and AMPK is not activated. Under pathological conditions, active classic RAS (ACE1/AngII) leads to an increase in AngII, and AngII inhibits AMPK through AT1R. After AMPK is inhibited, ACE expression is enhanced and the classical pathway is further enhanced. The expression of ACE2 and MasR is weakened, and the protective arm is (ACE2/Ang(1-7)) further inhibited. When the RAS guard arm (ACE2/Ang(1-7)) is boosted, AMPK is activated and the classical RAS pathway is inhibited. Similarly, when AMPK is activated, the RAS protection arm is enhanced, the classical RAS pathway is inhibited, and the RAS balance is promoted this process may involve other factors such as sirtuins, adiponectin, and PPARs. Besides, the activated AMPK can improve the RAS classical pathway-induced damage by adjusting its downstream cellular signaling pathways. RAS and AMPK have very close associations. Precise cooperation of RAS and AMPK is necessary for the efficient regulation of homeostasis (Figure 2).

In recent years, the benefits of blocking the classical RAS pathway for cardiovascular disease has been widely recognized, and even large-scale clinical trials have been conducted using both the ACEI and ARB dual-blocking classical pathways, but the results show that no more benefits are obtained. With the related research on the RAS protection pathway and the more profound understanding of RAS and AMPK correlation, the combination of blocking the classical pathway of RAS, strengthening the protective arm, and activating AMPK might gain more benefits. To better understand the link between the two factors, more animal experiments and clinical research are needed.

## Abbreviations

ACE: angiotensin-converting enzyme
ACEI: angiotensin-converting enzyme inhibitors
Agt: angiotensinogen
AICAR: 5-aminoimidazole-4-carboxamide -1-β-d-ribofuranoside
AMPK: AMP-activated protein kinase
AngI: angiotensin I
AngII: angiotensin II
ARB: angiotensin II receptor blockers
ATP: adenosine triphosphate
AT1R: angiotensin type 1 receptor
AT2R: angiotensin type 2 receptor
CaMKKβ: Ca^2+^-/calmodulin-dependent protein kinase β
Cdk4: cyclin-dependent kinase 4
EAT: epicardial adipose tissue
eNOS: endothelial nitric oxide synthase
Epac: exchange protein directly activated by cAMP
ERK: extextracellular signal-regulated kinase
FKN: proinflammatory chemokine fractalkine
FOXO1: forkhead box protein O1
GLP-1: glucagon-like peptide-1
GLUT: glucose transporter
HFD: high-fat diet
HUVEC: human umbilical vein endothelial cell
LKB1: liver kinase B1
MasR: Mas receptor
MEK: extracellular signal-regulated kinase kinase
MrgD: Mas-related G-protein-coupled receptor, member D
OLETF: Otsuka Long Evans Tokushima Fatty
PAEC: pulmonary artery endothelial cell
PH: pulmonary hypertension
PPAR: peroxisome proliferator-activated receptor
PRR: prorenin receptor
P70S6K: p70s6 kinase
RAS: renin–angiotensin system
Rb: retinoblastoma protein
ROS: reactive oxygen species
SHR: spontaneously hypertensive rat
SIRT1: sirtuin 1
WT: wild-type
